# Benzodiazepine prescribing patterns among Medicare providers, 2017 to 2023

**DOI:** 10.3389/fmed.2026.1723651

**Published:** 2026-02-20

**Authors:** Olivia Silvernail, Alexis D. Ritvo, Bernard Silvernail, Peter R. Martin

**Affiliations:** 1 Alliance for Benzodiazepine Best Practices, Portland, OR, United States; 2 Department of Psychiatry, University of Colorado School of Medicine, Aurora, CO, United States; 3 Department of Psychiatry and Behavioral Sciences, Vanderbilt University Medical Center, Nashville, TN, United States; 4 Department of Pharmacology, Vanderbilt University Medical Center, Nashville, TN, United States

**Keywords:** benzodiazepine, long-term, Medicare, older adults, Part D, prescribing

## Abstract

**Introduction:**

To determine whether benzodiazepine (BZD) prescriptions in older adults (>65) generally adhere to the Beers Criteria of the American Geriatrics Society, and whether the likelihood of prescribing an average of > 30 days per beneficiary to older adults varies significantly, Medicare Part D prescribing patterns were analyzed based on prescriber location and specialty.

**Methods:**

The 2017–2023 Medicare Part D Prescriber Public Use and Summary files were evaluated, with drug event information aggregated by provider and type of drug. The most prescribed BZD and the most common prescriber specialty for each year was determined, as well as the drug utilization rate by state.

**Results:**

Medicare Part D BZD prescriptions rose from 1.7 million to 3.1 million, an *increase* of over 80%, between 2017 and 2023, while overall U.S. BZD prescriptions *decreased* by about one-quarter during this same period, from 110 million to 81 million. The top three most prescribed BZD medications were alprazolam, lorazepam, and clonazepam, totaling around 88% of all the BZD prescriptions being written to Part D beneficiaries. When adjusted for the number of prescribers, all classes of prescribers increased their use of BZDs. Psychiatry was the top prescribing specialty, and showed the most increase in BZD prescription rate. The southeast region of the U.S. had the highest BZD utilization rates, with approximately 25 prescriptions dispensed for every 100 beneficiaries. In 2023, psychiatrists were more likely to prescribe BZDs for >30 days compared to nurse practitioners, physician assistants, and providers in the ‘other’ category, but less likely than family practice, internal medicine, and geriatric medicine providers. Compared to California, significantly increased odds of prescribing >30 days were found in 13 U.S. states; significantly decreased odds occurred in 5 states. Despite guidelines recommending against prescribing BZDs to adults ≥ 65 years and limiting prescriptions to 30 days or fewer, all years studied exceeded these guidelines, averaging 108 days of BZD per beneficiary.

**Conclusion:**

Despite repeated warnings about the harms of their use in this population, BZDs are being increasingly prescribed to Medicare Part D recipients. Some states and prescriber classes prescribe BZDs significantly over 30 days per beneficiary to older adults.

## 1 Introduction

Benzodiazepines (BZDs) are a class of central nervous system depressant medications commonly prescribed for anxiety and insomnia. The Food and Drug Administration (FDA) added a boxed warning to BZD labeling in 2020 to alert prescribers that BZDs should not be prescribed for long-term use due to the risks of dependence, potential for tolerance, and counteracting effects such as worsening anxiety (1). While there is little convincing research supporting efficacy beyond four weeks of use, (2) roughly a third of all BZD use exceeds four months, with older adults accounting for the greatest proportion of use ≥ 120 days (3).

Four systematic reviews and meta-analyses have shown that long-term BZD use, that is use over 30 days, is associated with cognitive impairments including difficulty with sensory processing, problem-solving, concentration, motor control, and memory (4–7). While such impairments may improve upon BZD discontinuation, protracted effects have been reported in a subset of patients that endure after full discontinuation (8). Studies show that BZD use in older adult populations remains high and long-term use increases with age (3). Elderly populations are particularly vulnerable to the adverse effects of BZDs, due to associations with an increased risk of falls, fractures, and cognitive impairment which can lead to a significant increase in morbidity and mortality (9–13).

The Medicare Modernization Act excluded BZDs from Medicare Part D coverage from 2006 to 2012 due to their associated risk of increased falls and hip fractures in older individuals (14). In its authoritative Beers Criteria, the American Geriatrics Society lists BZDs as inappropriate medications for seniors outside of certain restricted uses: seizure disorders, rapid eye movement sleep behavior disorder, benzodiazepine withdrawal, ethanol withdrawal, severe generalized anxiety disorder, and periprocedural anesthesia (15), yet the proportion of patients who are prescribed long-term BZD therapy increases with advancing age (14.7% of long-term BZD users are 18 to 35 years of age, compared to 31.4% who are 65 to 80 years old) (3).

Previous studies have shown the prevalence of BZD use in older adults from 2010 through 2017 has been correlated to opioid use and patient education levels. However, as noted by Maust et al. in 2018, “within primary care, the links between geographic or prescriber characteristics and BZD prescribing have been subject to little research” (10, 11, 16–18). This situation remains unchanged as of this writing, which is our motivation for performing this study. The aim of this study was to examine recent BZD prescribing patterns and drug utilization rates for those ≥ 65 years and older in the United States. Data were analyzed based on geographical location, prescriber specialty, using provider-level prescribing rates and state-level prescription utilization rates.

## 2 Materials and methods

### 2.1 Dataset

The data used in this analysis were taken from the de-identified, aggregated public use files in the Medicare Part D Prescribers – by Provider and Drug dataset, provided by the Centers for Medicare and Medicaid Services (CMS), and therefore did not require institutional review board approval. The most recent seven years available (2017–2023) were used to analyze trends over time. It is important to note that the data from 2020 and 2021 could potentially be affected by the COVID-19 pandemic.

Medicare Part D provides prescription drug coverage for U.S. residents aged ≥ 65 or for younger individuals with qualifying disabilities. Our study was restricted to beneficiaries ≥ 65, which represent an older, more medically complex population compared to the general U.S. population and applies to the population addressed by the Beers Criteria.

The dataset contains aggregated claims data at the provider-drug level, including the number of beneficiaries receiving each medication and the total days supplied, but does not include beneficiary details such as diagnosis or clinical outcomes. The data reflects dispensed prescriptions paid by Medicare Part D, which captures pharmacy redemptions and does not account for prescriptions written but not filled, nor does it distinguish between continuous and as-needed prescriptions.

All analyses were run using SAS Studio (SAS Institute, Cary, North Carolina), while data visualizations were produced using Flourish Software (Canva, Sydney, Australia).

### 2.2 Population

Providers included in this study practiced in the United States (50 states and Washington D.C.) based on the prescriber’s practice location as listed on the Medicare Part D Prescriber file. Prescribers in United States territories were excluded due to Medicare Part D coverage differing in territories, particularity regarding enrollment patterns, and is not directly comparable for the purpose of analyzing geographic variation. Prescribers were included if they had at least one prescription claim for a beneficiary aged ≥ 65 years in the calendar year for one of the following BZDs: alprazolam, chlordiazepoxide, clobazam, clonazepam, clorazepate, diazepam, estazolam, flurazepam, lorazepam, midazolam, oxazepam, quazepam, temazepam, and triazolam. These are the only BZDs that are FDA approved for prescription in the U.S. on an outpatient basis, as listed in a review of best practices in BZD prescribing (19).

CMS suppresses beneficiary counts for providers who prescribed a particular medication to fewer than 11 beneficiaries to protect patient anonymity. As a result, the analysis was restricted to the unsuppressed subset, representing 13.1% of total Medicare Part D BZD prescribers. This restriction may introduce selection bias toward higher-volume prescribers and limits generalizability.

**Figure 1:**
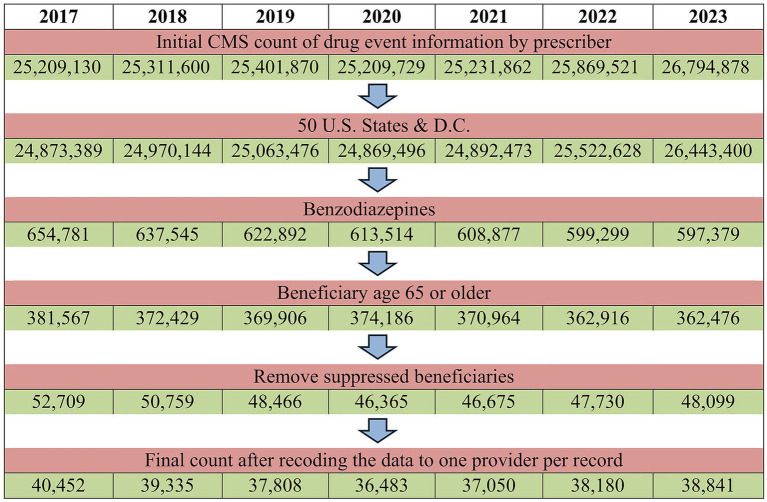
Number of records remaining after each step of data filtration, shown for each year of data used. The final count is the number of providers that had 11 or more filled prescriptions for a specific BZD in that year.

To prepare for analysis, the dataset was recoded to one record per provider-beneficiary combination per year. The final dataset after these exclusions was comprised of 7 years of provider observations, ranging from 40,452 unique providers in 2017 to 38,841 in 2023 (See Figure 1).

### 2.3 Provider demographics

Providers were grouped by specialty and provider types. The CMS taxonomy includes separate categories for psychiatry, psychiatry-neurology, and neurology. For this analysis, the three were combined into a single ‘psychiatry’ grouping because the psychiatry-neurology group spans elements of both specialties. BZD prescribing patterns may differ between these groups, which is a limitation of the grouping.

Other specialty categories included family practice (includes general practice), internal medicine, geriatric medicine, and “other.” An “other” category was created for specialties not included elsewhere, which individually accounted for < 1% of prescriptions written. Although geriatric medicine prescribed < 1% of all prescriptions, it was included as its own category because it specializes in the care of the population of interest. Nurse practitioners and physician assistants were included as provider types but were not categorized by specialty.

### 2.4 Definition of prescription duration categories

A 2023 scoping review of all known BZD prescription guidelines showed that most recommended use only when necessary, for either <4 weeks or for an unspecified “short-term” use duration (20). Several other studies and a 2023 BZD tapering guideline classify long-term use as >4 weeks and recommended against long-term use (21–24). A recently released BZD tapering guideline classifies >120 days use in a year as “long-term” and warns that “physical dependence can develop within weeks” (25). The FDA 2020 boxed warning noted above did not formally define “long-term” (1). Faced with this conflicting information, and for ease of analysis, this study chose to follow the majority of sources and define “short-term” as ≤ 30 days supplied per beneficiary per year, calculated from aggregated days-supplied fields.

### 2.5 Statistical analysis

The Medicare Part D Prescribers – by Provider and Drug dataset does not contain beneficiary-level claims. For each provider and drug in a given year, CMS reports the total number of prescription claims, the total days supplied across all filled prescriptions, and the number of beneficiaries who received that medication from that prescriber.

Using these aggregated fields, the mean number of BZD days supplied per beneficiary per year per prescriber was determined. This is called *days_filled* and was calculated for a given year as the total days supplied for all BZD prescriptions divided by the number of beneficiaries receiving a BZD from a particular provider.

The most frequently prescribed BZDs and most common prescriber specialty and prescriber types were identified based on claim frequency. Prescribing rate was defined as the mean number of BZD claims within a specialty divided by the number of prescribers in that group for each year.

The data were aggregated based on state location to find the total number of BZD prescription claims dispensed per state. This information was then used to calculate the utilization rate (BZD claims per 100 Part D beneficiaries) in each state based on yearly totals of Medicare beneficiaries enrolled in Part D coverage per state, based on data collected by the Kaiser Family Foundation (26). Drug utilization rates per state were categorized into quintiles and mapped geographically using Flourish data visualization software (Canva, Sydney, Australia). The quintiles ranged from 0 to 26 prescriptions per 100 beneficiaries, based on the lowest and highest rates seen over all 7 years.

Providers were categorized into a binary variable, with 0 being an average of ≤ 30 days of medication per beneficiary per year, and 1 being an average of > 30 days per beneficiary per year (hereafter referred to as “≤ 30 days” and “>30 days,” respectively). A type III Wald chi-squared test was run for each year to determine if the relationship between over/under guideline prescribing, geographic location, and prescriber specialty was significant. Then, a logistic regression model was used to model the probability of guideline prescribing in each state, after adjusting for the provider’s specialty, as well as the probability of guideline prescribing for a given specialty after adjusting for the prescriber’s state. California was used as the state reference level for the logistic regression, as it had the highest overall number of prescribers. Psychiatry was used as the specialty reference level since this group specializes in prescribing anxiolytic medications. The final model was evaluated using a Hosmer-Lemeshow (H-L) goodness of fit test.

## 3 Results

**Figure 2:**
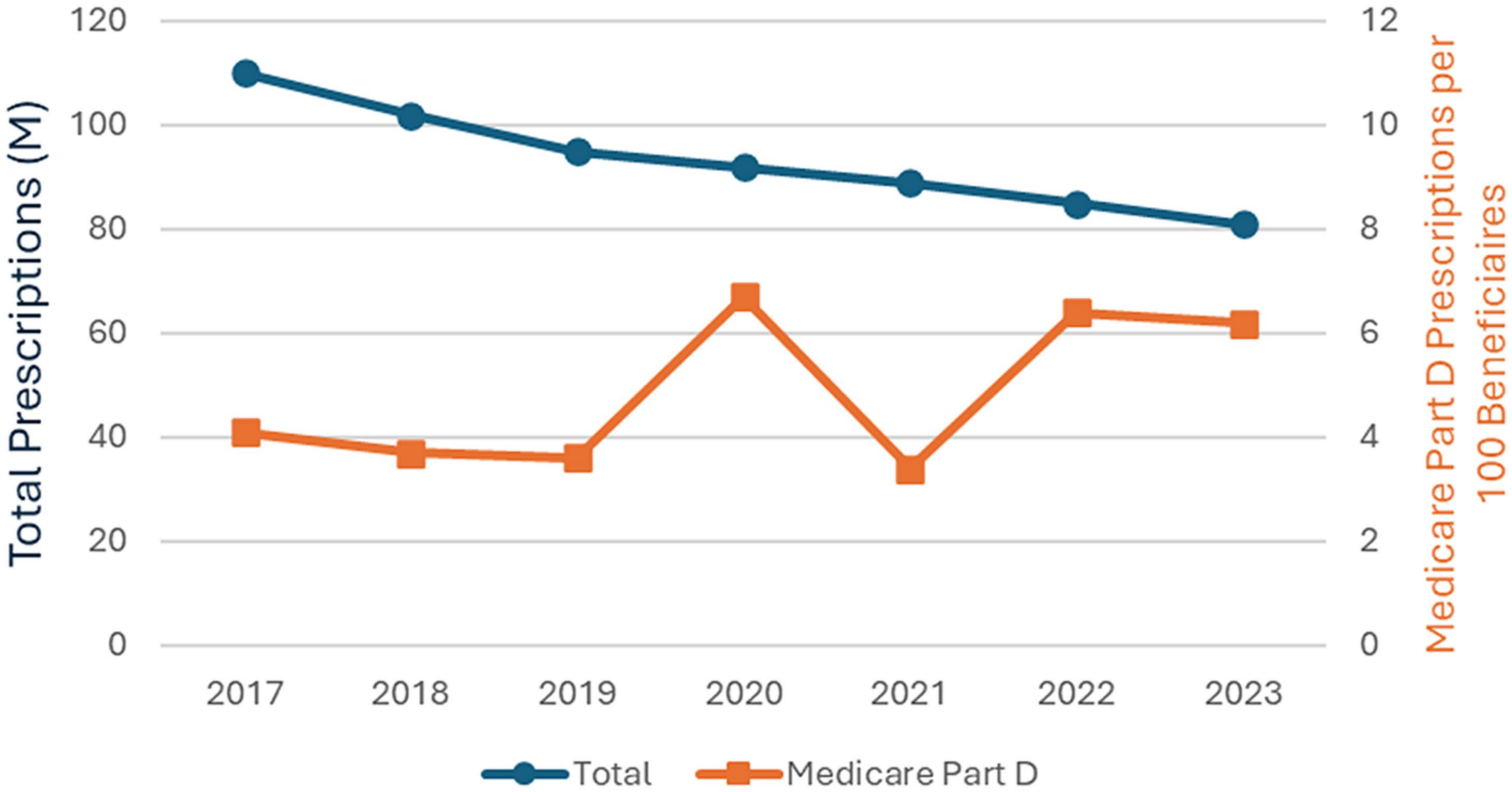
Variation of BZD prescriptions for the total U.S. and Medicare Part D, 2017–2023. The Total line represents U.S. prescription across all ages. The Medicare Part D line represents prescriptions dispensed per 100 Medicare Part D beneficiaries aged ≥ 65, accounting for enrollment changes over time. Note the different axes for the two plots.

During the study period overall U. S. BZD prescriptions decreased steadily during the study period, from 110 million to 81 million, a net reduction of about one-quarter (27). In contrast, when normalized to Medicare Part D enrollment, BZD prescribing among Part D beneficiaries increased by approximately 51%, despite the overall decrease in national prescribing (Figure 2).

**Figure 3:**
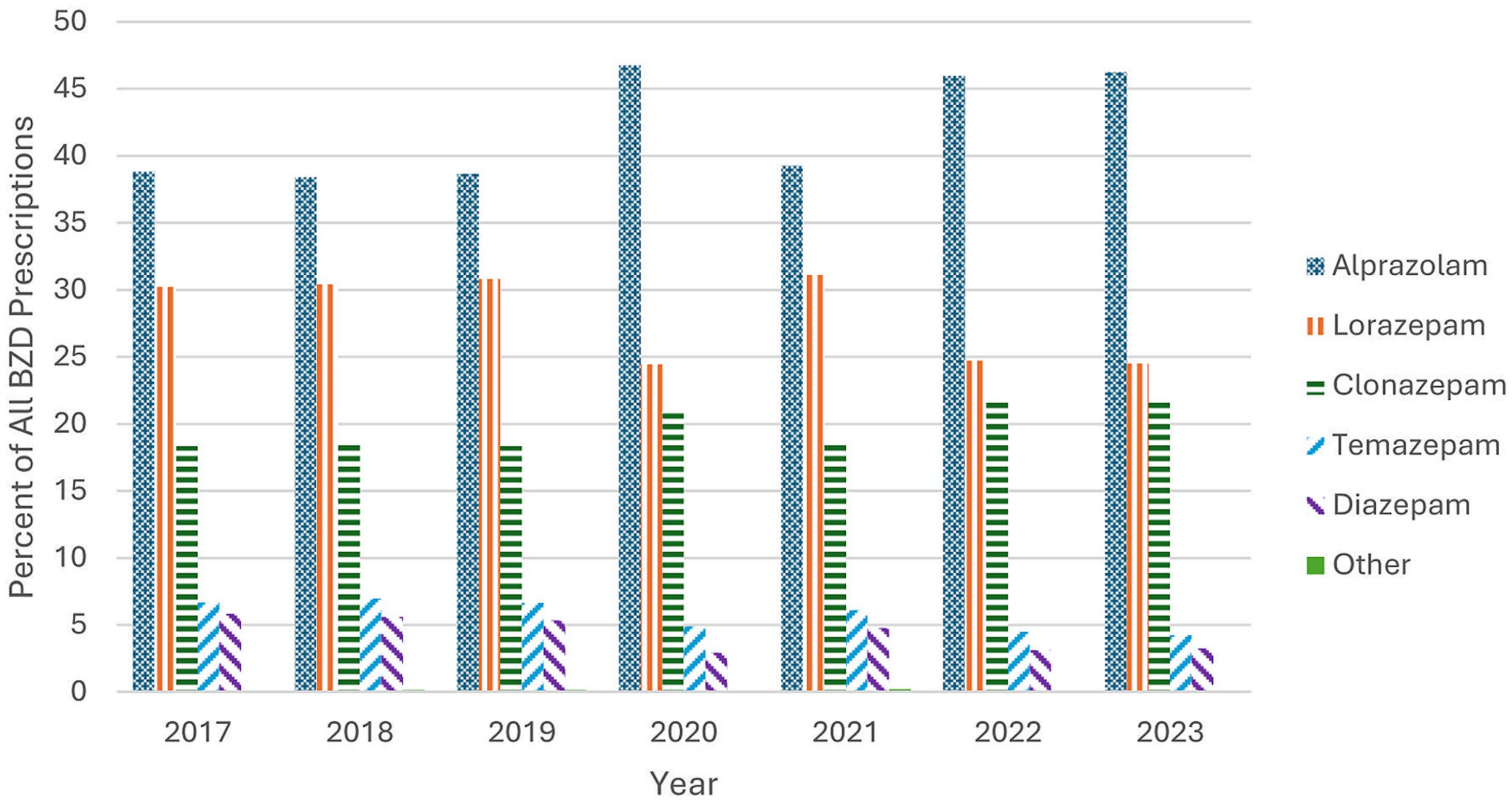
Top BZD prescriptions based on yearly percentage. The most frequently prescribed BZDs are ranked in order of number of claims by year studied.

As shown in Figure 3, the order of the most-prescribed BZDs remained unchanged over the study years, with alprazolam consistently the most frequently prescribed BZD, ranging from 38.4 to 46.8% of all Medicare Part D BZD prescriptions. The upward trend in alprazolam and clonazepam align with the increase in overall Medicare Part D BZD prescriptions shown in Figure 2.

This study found a mean of 108 days and median of 103 days of medication were authorized to beneficiaries.

**Figure 4:**
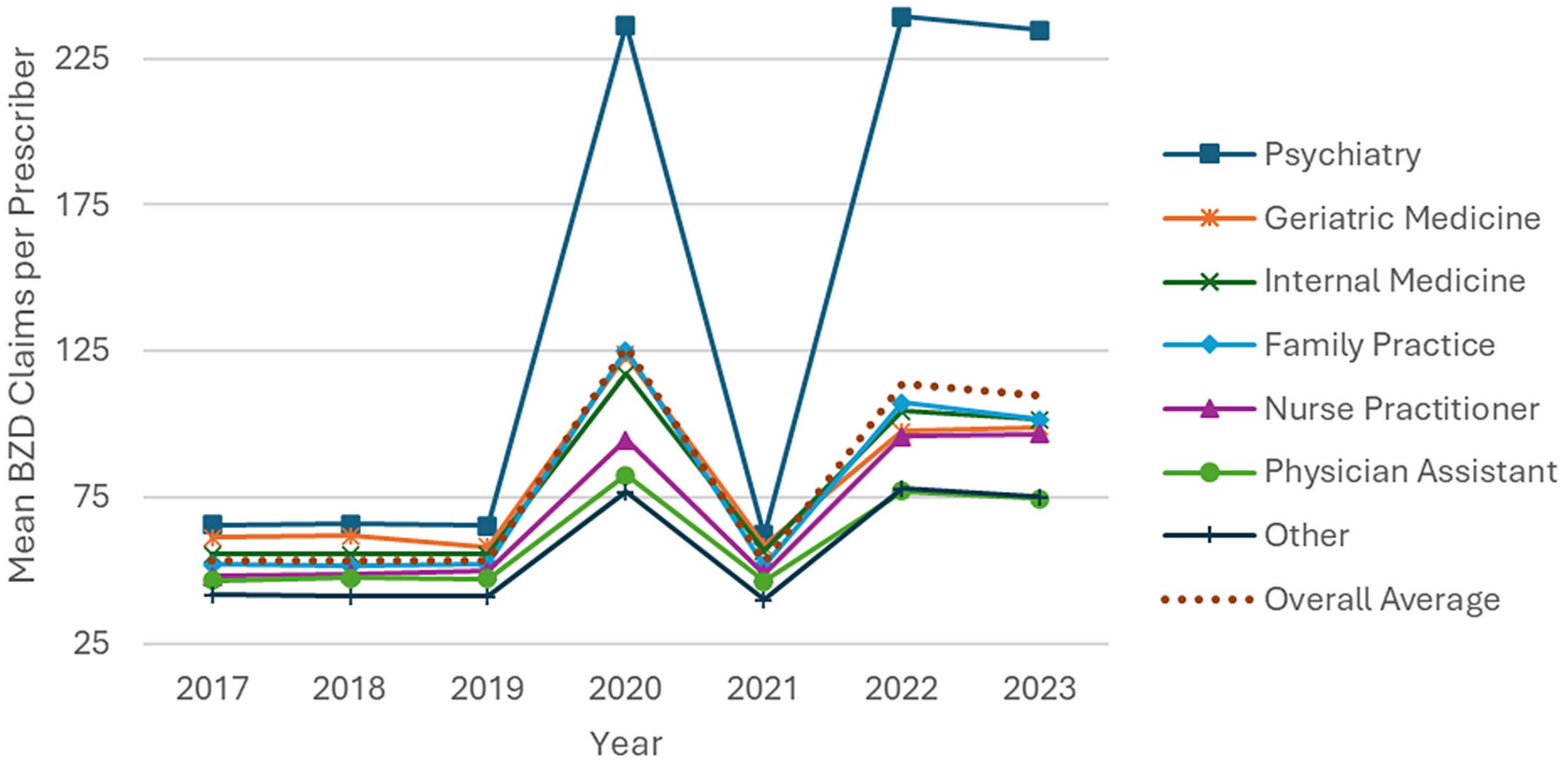
Prescribing rate (mean BZD claims per prescriber per year), by specialty. The vertical axis represents the average number of benzodiazepine claims written per prescriber within each specialty group.

The prescribing rates of the various prescriber classes are shown in Figure 4. Since the number of clinicians in each class varies throughout the study period, BZD prescribing rate is reflected as the mean prescription claims per prescriber type per year.

Four groupings stand out over the study period. First, in all years psychiatrists had the highest prescribing rate, roughly four times the overall average in most years, and rising from about 66 claims per prescriber in 2017 to 235 in 2023 (an increase of ~250%). Second, family practice, internal medicine and geriatric medicine specialists plus nurse practitioners all increased prescribing, with rates roughly doubling across the study period. These groups closely tracked the overall average and together represent approximately 3/4 of all Medicare Part D prescribers. Third, physician assistants increased their prescribing from 47 to 75 claims per prescriber, had the lowest rate of increase at about 60%, and remained below the overall average throughout. Fourth, the “other” category consistently had the lowest prescription rate (varying from 42 to 75 claims per prescriber), and had among the smallest increase in prescribing, about 80%. All classes of prescribers saw the same prescription pattern over the study period, but to different degrees.

**Figure 5:**
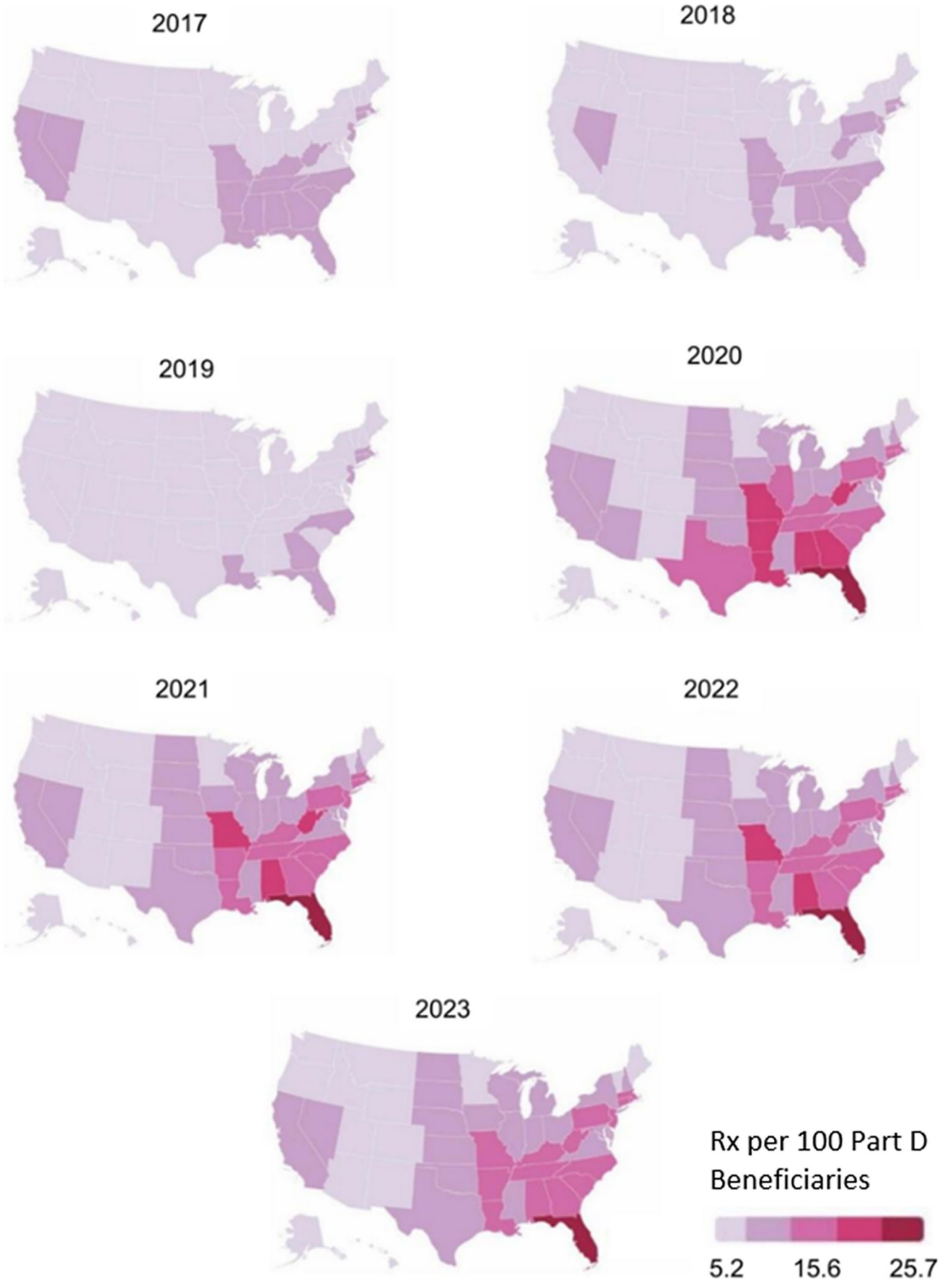
BZD utilization rates by geographical location in the United States and by year.

Figure 5 shows the BZD utilization rates across the U.S. for each year. These maps represent the number of BZD prescriptions (fills) dispensed per 100 Medicare Part D beneficiaries in each state, which accounts for differences in Part D enrollment. From 2017 to 2019, utilization rates decreased across the U.S., with all states dropping into the lowest two quintiles by 2019. One year later, the southeast region increased in utilization rates from approximately 8 to 25% of Part D beneficiaries receiving a BZD prescription. Overall BZD utilization decreased from 2021 to 2023, with the highest use remaining in the southeast.

According to the Type III chi-squared test for the multicategory variables of provider state and provider specialty or type, the relationship between provider specialty or type and >30 day average prescribing (Table 1, *p*-value<0.001) and the relationship between provider state and >30-day average prescribing (Table 2, *p*-value<0.001) is significant. Based on these statistically significant results, we then conducted a logistic regression to explore how provider specialty and state influenced the likelihood of prescribing >30 days. Across all years, the proportion of providers prescribing > 30 days remained consistently high and relatively stable (87.9–95.6%).

**Table 1:** Association between prescriber characteristics and >30-day average prescribing of benzodiazepines in 2023.

Specialty or type	Odds ratio	95% Confidence interval	*p*-value	n (prescribers)
Internal medicine	1.885	1.636–2.171	<0.0001	11,572
Family practice	1.842	1.596–2.197	<0.0001	10,104
Geriatric medicine	1.714	1.047–2.805	0.0321	312
Psychiatry	*reference*	*reference*	*reference*	4,139
Nurse practitioner	0.725	0.634–0.830	<0.0001	7,060
Physician assistant	0.441	0.376–0.518	<0.0001	2,075
Other	0.086	0.075–0.098	<0.0001	3,579

**Table 2:** Association between state and >30-day average prescribing of benzodiazepines in 2023.

State	Odds ratio (95% CI)	*p*-value
Alabama	1.141 (0.844–1.544)	0.3907
Alaska	0.735 (0.255–2.121)	0.5691
Arizona	0.886 (0.686–1.145)	0.3563
Arkansas	1.277 (0.869–1.878)	0.2127
California	*reference*	*reference*
Colorado	0.469 (0.358–0.613)	<0.0001
Connecticut	1.645 (1.227–2.206)	0.0009
District of Columbia	0.499 (0.208–1.196)	0.1190
Delaware	0.943 (0.561–1.584)	0.8239
Florida	1.430 (1.236–1.655)	<0.0001
Georgia	0.907 (0.739–1.114)	0.3528
Hawaii	0.899 (0.438–1.844)	0.7714
Idaho	1.405 (0.735–2.685)	0.3038
Illinois	1.647 (1.313–2.066)	<0.0001
Indiana	0.860 (0.665–1.111)	0.2479
Iowa	1.729 (1.139–2.623)	0.0101
Kansas	1.427 (0.920–2.213)	0.1121
Kentucky	0.986 (0.732–1.328)	0.9262
Louisiana	1.545 (1.151–2.076)	0.0038
Maine	0.552 (0.358–0.851)	0.0072
Maryland	1.116 (0.828–1.504)	0.4709
Massachusetts	1.482 (1.190–1.845)	0.0004
Michigan	0.880 (0.714–1.085)	0.2323
Minnesota	0.862 (0.612–1.214)	0.3945
Mississippi	0.964 (0.621–1.498)	0.8717
Missouri	0.879 (0.683–1.131)	0.3152
Montana	1.130 (0.545–2.341)	0.7424
Nebraska	1.847 (1.048–3.255)	0.0339
Nevada	1.262 (0.841–1.896)	0.2615
New Hampshire	0.688 (0.464–1.021)	0.0634
New Jersey	2.567 (2.038–3.233)	<0.0001
New Mexico	0.892 (0.503–1.582)	0.6966
New York	1.529 (1.286–1.818)	<0.0001
North Carolina	1.136 (0.931–1.387)	0.2076
North Dakota	0.563 (0.289–1.100)	0.0926
Ohio	0.810 (0.663–0.990)	0.0399
Oklahoma	0.889 (0.612–1.293)	0.5388
Oregon	0.417 (0.309–0.561)	<0.0001
Pennsylvania	1.421 (1.176–1.718)	0.0003
Rhode Island	2.031 (1.181–3.490)	0.0104
South Carolina	0.955 (0.729–1.251)	0.7398
South Dakota	0.996 (0.518–1.915)	0.9897
Tennessee	1.400 (1.072–1.827)	0.0134
Texas	1.395 (1.168–1.666)	0.0002
Utah	1.120 (0.667–1.880)	0.6691
Vermont	1.425 (0.578–3.510)	0.4415
Virginia	1.024 (0.791–1.326)	0.8579
Washington	0.420 (0.319–0.553)	<0.0001
West Virginia	1.394 (0.842–2.309)	0.1965
Wisconsin	1.046 (0.789–1.387)	0.7556
Wyoming	1.731 (0.358–8.376)	0.4949

Statistically significant outcomes are indicated by yellow shading.

The logistic regression model used data from 2023, the most recent year for which data was available. Reference levels were set: California for the analysis by state, and Psychiatry for the analysis by specialty or type. The specialty or type of prescriber regression (Table 1) showed that patients prescribed BZDs by nurse practitioners (OR 0.725, 95% CI 0.634–0.830), physician assistants (OR 0.441, 95% CI 0.376–0.518), or “other” specialists (OR 0.086, 95% CI 0.075–0.098) were less likely to be prescribe BZD > 30 days compared to those treated by psychiatry. In contrast, family practice (OR 1.842, 95% CI 1.596–2.197), internal medicine (OR 1.885, 95% CI 1.636–2.171), and geriatric medicine (OR 1.714, 95% CI 1.047–2.805) practitioners had increased odds of prescribing >30 days. The number of prescribers in each group ranged from 312 (geriatric medicine) to 11,572 (internal medicine), and confidence intervals were generally narrow except for geriatric medicine, reflecting the smaller sample size in this specialty.

The regression results also showed evidence that, after adjusting for prescriber specialty or type, patients in 13 states (see Table 2) had significantly higher odds of being prescribed BZDs >30 days compared to those in California (*p* < 0.05). In contrast, patients in Colorado, Maine, Ohio, Oregon, and Washington are associated with a decreased risk. The Hosmer-Lemeshow goodness-of-fit test did not indicate evidence of poor model fit (*p* = 0.0634).

## 4 Discussion

Except for a few specific cases, BZD use is not recommended for adults aged ≥ 65 years due to its association with an increased number of falls, fractures, and cognitive impairment (15). When BZDs are used, exposure > 30 days is not recommended because of its lack of studied efficacy and risk of developing dependence (19, 20). This study found an average of 108 days per beneficiary were dispensed, which far exceeds the 30 day recommended maximum. Therefore, these recommendations are not being followed for Medicare Part D beneficiaries, who have been shown to be more susceptible to harmful effects of the medication.

The Chi-squared test found a significant relationship between provider state and prescribing patterns (>30-day average per beneficiary), as well as a significant relationship between provider specialty or type and prescribing patterns. The results of the logistic regression, after adjusting for provider location, indicated that prescriptions written by a nurse practitioner, physician assistant, or providers in the “other” specialty category had significantly lower odds of prescribing BZD > 30 days compared to psychiatrists. Physician’s assistants and the “other” specialty category had the lowest and third-lowest rates of BZD prescription increase, at 60 and 80%, respectively, while nurse practitioners nearly doubled their rate. These groups are prescribing more BZDs to Medicare Part D patients, but they are prescribing them for shorter time periods, moving them in the direction of aligning with the Beers Criteria.

Providers in family practice and internal medicine had significantly higher odds of prescribing >30 days, and their rate of prescription increased by 94% for family practice and 83% for internal medicine over the study period. This led these large groups to have among the highest prescribing rates by the end of the period, exceeded only by psychiatrists. This suggests there is a significant problem with adherence to the Beers Criteria among family and internal medicine practitioners.

Geriatricians, averaging only 0.8% of the prescriber population, also had significantly higher odds of prescribing >30 days, but their rate of prescription increased by the second-lowest rate, 60%.

Psychiatrists, the reference group for probability of prescribing > 30 days, had the highest prescribing rate, which also increased at the highest rate throughout the review period. Compared with psychiatrists, internal medicine, family practice and geriatrics all had higher rates of prescribing >30 days, while nurse practitioners, physician assistants and the “other” group all had lower rates of prescribing >30 days. One possible explanation for this high and increasing rate of prescription is that many of the other clinician types have reduced their use of BZDs in this population by either referring the more difficult or persistent cases to other caregivers, or by simply refusing to further prescribe BZDs to these patients. Psychiatrists are often the best-trained specialists to treat patients with long-term physiological dependence on BZDs, and wind up “inheriting” these legacy patients and therefore are tasked with stewardship of BZDs and attempting to discontinue these medications as clinically appropriate.

In this context, it must be considered that nurse practitioners and other clinicians, such as those in general or family practice, often find themselves on the “front lines” of mental health care. While they are likely to consult with many patients with a range of common mental health problems, such as depression and anxiety, patients with more severe forms of mental illness or those with complex cases and mental health comorbidities are more likely to be treated by psychiatrists. Thus, nurse practitioners and other clinicians are likely to see more patients, but patients with short-term or less severe problems, than psychiatrists, who are likely to see fewer patients but with more challenging cases. It is possible that these more complex cases require more BZDs, but this merits further study.

All classes of Medicare Part D prescribers followed a similar BZD prescription pattern between 2017 and 2023. Figure 4 shows that prescription approximately doubled in 2020, decreased to 2019 levels in 2021, then increased to 2020 levels for the next 2 years. One possible explanation for part of the increase in 2020 is the stress and disruption of the COVID-19 pandemic, and the reduction in 2021 may be partially due to decreased patient contact during the height of infections, which may include a long-term increase in the use of BZDs (28–30). The overall trend of increasing use in this population may also be at least partially attributed to the aging of a population increasingly dependent on BZDs. Note that these results are in the opposite direction of studies conducted on 2010 to 2017 data (17, 18).

Alprazolam, clonazepam, and lorazepam were the three most-prescribed BZDs, comprising over 89% of all BZD prescriptions. Alprazolam and lorazepam are both short-acting medications, while clonazepam is a long-acting BZD, and both alprazolam and clonazepam prescriptions in this population are increasing, despite the national trend in the opposite direction. In any case, the high rates of BZD prescribing in light of warnings about dependence and withdrawal issues is concerning.

Nationwide, Medicare Part D BZD utilization declined from 2017 to 2019, followed by a noticeable increase in the southeastern U.S. beginning in 2020. This upward trend persisted through 2022, with a slight decline observed in 2023.

The results of the logistic regression, after adjusting for provider specialty, showed 13 states with significantly higher odds of BZD prescribing >30 days compared to California (all *p* < 0.05; Table 2). Notably, providers in Connecticut, Illinois, Iowa, Louisiana, Nebraska, New Jersey, New York, and Rhode Island had odds ratios above 1.5, indicating that patients who had a BZD prescription written in these states had at least 1.5 times the odds of being prescribed >30 days. The most extreme case was New Jersey, where the odds were more than 2.5 times higher (OR = 2.567, *p* < 0.0001). On the other hand, Colorado, Maine, Ohio, Oregon, and Washington had significantly lower odds of >30-day prescribing in 2023 (all *p* < 0.05). Whether this scope of practice factor is meaningful to understand BZD prescribing in older adults requires further study. These outliers also could be the result of many factors, including income, accessibility to health care, legislation and regional attitudes.

Our study did not include the possible effects of population changes among American regions, which might also have played a role. It is not clear how regional changes might affect global prescribing patterns, but such geographical distinctions apply across the U.S. as well. These regional differences suggest that a geographically bounded prescribing culture may exist (31). Some regions may have notable obstacles to referring patients to psychiatric care. Other higher-prescribing regions may lack a strong interdisciplinary approach to medicine, have limited resources to manage patients compared to wealthier areas, and have weak or limited pharmacist reviews. Since tapering BZD to discontinuation can be a long, arduous, and difficult process, a lower rate of tapering may contribute to the regional differences noted (32). These geographical differences are worth further examination, as they may help provide insight in terms of how to shift prescribing practices. The term “prescribing culture” is coined for this article (and differs from “culture-based prescribing”) but may turn out to be a serviceable distinction in better understanding BZD prescribing patterns. Further study is warranted.

This study has several limitations. The Medicare Part D dataset only includes providers with more than 10 total claims and only includes drugs for which an individual prescriber has more than 10 claims (33). While this protects the privacy of beneficiaries, it underestimates the Part D totals. Findings may therefore not be generalizable to clinicians who prescribe BZDs infrequently, and it is not possible to determine whether suppressed prescribers have higher or lower prescribing rates per patient than those included in the analysis.

Additionally, these data are limited to only information from Medicare beneficiaries with Part D coverage, thus excluding those Medicare beneficiaries without prescription coverage and older adults served by the Veteran’s Administration system. The data is also aggregated at the prescriber level, therefore it is not possible to assess continuous versus as needed use, clinical indications, dosing regimens, treatment outcomes, or patient panel size, which limits inference about individual provider prescribing behavior. Since the study totals the number of days of prescription per year per provider, multiple <30-day prescriptions may net > 30 days. Regression analyses were limited to prescriber specialty and geographic location and did not contain patient-level characteristics due to limitations of the dataset. Therefore, the regression results should be interpreted as descriptive associations rather than causation. Finally, our study did not account for all variables which may have played a role in BZD prescribing, such as regional differences in education and income, comorbidities, age, and the healthcare environment by state, such as whether the state’s healthcare system includes large teaching institutions.

There is no widely recognized BZD prescribing guideline, and the 92 US and international guidelines identified by Brandt et al. vary significantly in their recommendations (20). Educational efforts have been only partially successful in changing BZD prescribing patterns. It may be that other means must be utilized, such as prescription drug monitoring programs or overprescription warnings to prescribers in electronic health records. Patient education and greater health literacy in the population may also be useful.

BZDs are generally not indicated for > 4-week use in any patients and are considered inappropriate for most geriatric patients. Obviously, medicine is not a one-size-fits-all science and there will be exceptions to these rules, but exceptions by definition should be rare. This study found that among clinicians who prescribed BZDs to more than 10 people ≥ 65 years of age, they prescribed for an average of 108 days per beneficiary. The geographical disparities in prescribing practices are striking and needs further study. Prescribing patterns do differ for other drugs, such as antibiotics (34), and these differences have been described as “cultural” (35). The American healthcare system is characterized by variations in how healthcare is provided, whether in small private practices or large teaching hospitals, the prevalence of specific comorbidities by region, healthcare disparities such as income and race, and the local level of healthcare literacy which, in turn, drives expectations from healthcare providers (35). The emergence of internet-driven continuing medical education and hopefully future national BZD prescription guidelines may reduce these disparities.

## 5 Conclusion

This study used the Medicare Part D dataset to demonstrate that BZDs are not only widely prescribed to those >65, but they are also often prescribed in direct contradiction to the Beers Criteria, prevailing guidelines and medical understanding. BZDs are recommended primarily for short-term use and with a few exceptions, are not appropriate for geriatric patients, yet they are often prescribed long term and are frequently administered to this population. While BZD prescription rates have fallen nationally, all classes of clinicians have increased their use among this vulnerable population. Regional differences in prescribing patterns also emerged. Psychiatry was the top prescribing specialty and showed the most increase in BZD prescription rate, but was less likely than family practice, internal medicine, or geriatric medicine to prescribe for >30 days. Nurse practitioners are prescribing more BZD than in the past, but they are also more likely to see patients with mental health conditions than in prior years owing to changes in practice. Overall, a consistent nationwide BZD prescribing guideline needs to be developed.

## Data Availability

Publicly available datasets were analyzed in this study. This data can be found: Centers for Medicare & Medicaid Services (CMS) Medicare Part D Prescribers - by Provider and Drug Public Use File (RRID: SCR_003962) https://data.cms.gov/provider-summary-by-type-of-service/medicare-part-d-prescribers/medicare-part-d-prescribers-by-provider-and-drug.
